# A Rare Case of Bilateral Orbital Metastases From Rectal Adenocarcinoma

**DOI:** 10.7759/cureus.29936

**Published:** 2022-10-05

**Authors:** Quratulain Khan, Hira Farooq, Alishbah Ziad, Anis Rehman, Kashif Siddique

**Affiliations:** 1 Radiology, Shaukat Khanum Memorial Cancer Hospital and Research Centre, Lahore, PAK

**Keywords:** palliative radiotherapy, periorbital pain, adenocarcinoma, rectum, orbital metastasis

## Abstract

Colorectal carcinoma is among the commonest malignancies in the World. However, metastases from rectal carcinomas to the orbit are extremely rare. Only a few such cases have been reported in the literature till date. We report a case of adenocarcinoma of the rectum in a 27-year-old male with bilateral orbital metastases who is currently undergoing palliative radiotherapy. Our aim is to highlight the role of imaging in the diagnosis and management of orbital metastatic disease.

## Introduction

Intraorbital metastases from distant organs are fairly uncommon. These can occur secondary to breast carcinoma, lung carcinoma, melanoma and prostatic carcinomas [1]. Although colorectal cancer is the third most common cancer in the world, it is extremely rare for it to metastasize to the orbital region, with only a few such cases being reported in the literature [2]. Twenty percent of colorectal carcinomas have already metastasized at the time of diagnosis, whereas nearly 30% metastasize afterward [3].

We report a case of bilateral orbital metastases in a young male patient with biopsy-proven adenocarcinoma of the rectum.

## Case presentation

A 27-year-old male presented to our hospital with complaints of per rectal bleeding and pain in the perianal region. Upon radiological and endoscopic investigations, he was found to have rectal growth. Biopsy of the rectal mass showed moderately differentiated adenocarcinoma. He underwent neoadjuvant chemo-radiotherapy followed by surgery. One year after surgery, he developed hepatic and pulmonary metastases. Despite palliative chemotherapy, his disease progressed.

**Figure 1:**
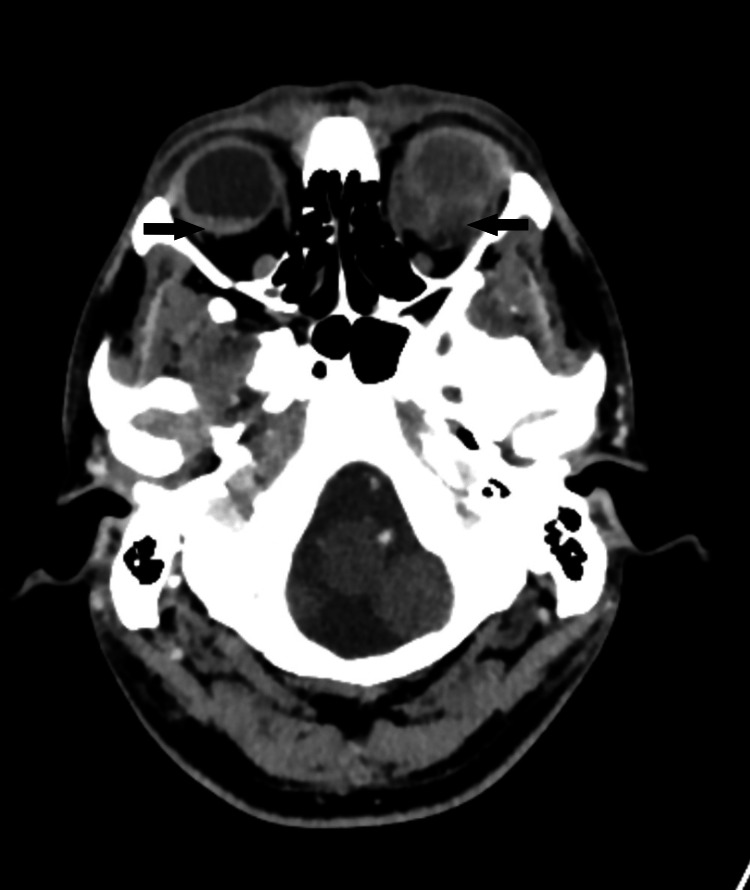
Contrast CT brain at the level of orbits demonstrates soft tissue masses involving bilateral globe (black arrows).

He later on presented to the emergency department with complaints of headache, decreased vision, and left periorbital region pain. A contrast CT brain was subsequently performed that demonstrated soft tissue masses involving bilateral globes (Figure 1).

**Figure 2:**
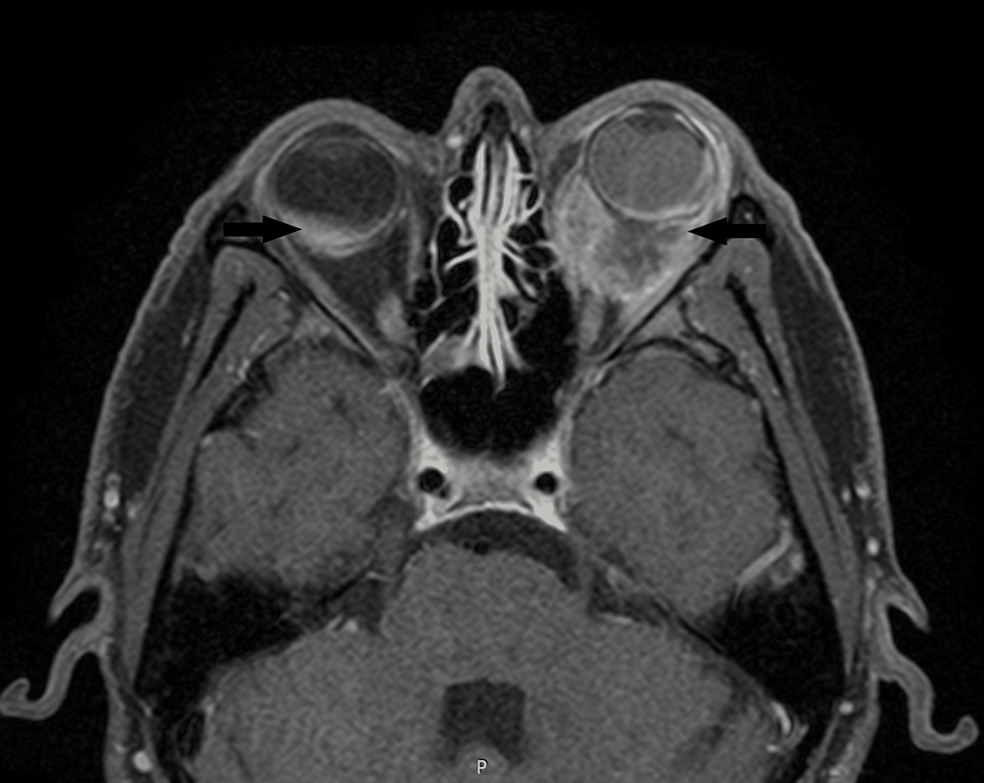
Plaque-like enhancing lesion in the posterior chamber of the right globe. Enhancing left intraconal mass extending into sclera of left globe is also seen (black arrows).

**Figure 3:**
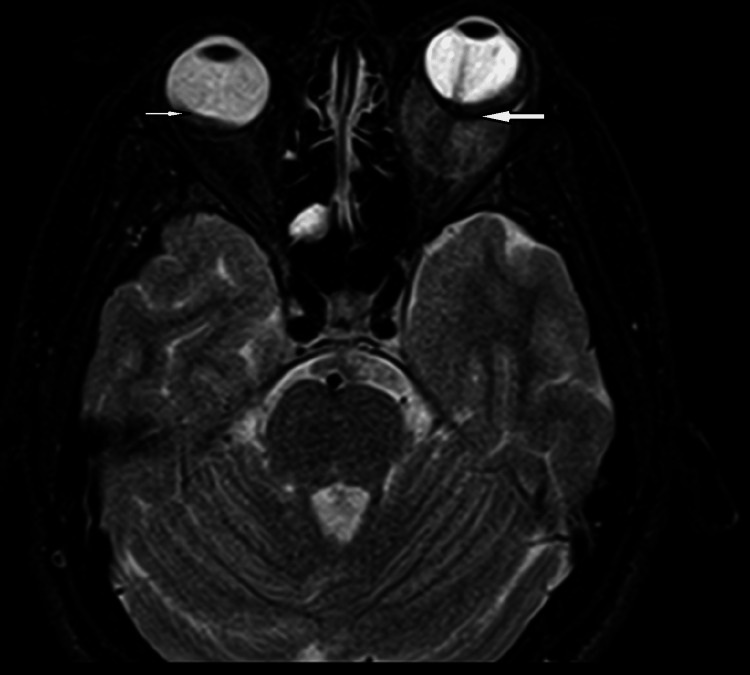
Bilateral orbital masses (white arrows).

CT was followed by MRI brain and orbits to obtain further soft tissue details and extension of the disease. MRI brain showed a plaque-like enhancing lesion in the posterior chamber of the right globe along with enhancement of the right sclera, choroid, and posterior globe, with corresponding diffusion restriction. Whereas, there was enhancing left intraconal mass showing contiguous extension in the sclera, choroid, posterior globe and intraconal segment of the left optic nerve (Figure 2-3). This mass measured approximately 25 x 20 x 17 mm in size.

The patient is currently undergoing palliative radiotherapy for the orbital metastatic lesions.

## Discussion

Metastatic orbital tumors range between 2% and 7% in various literature reviews [1,4]. On the other hand, orbital metastases originating from the gastrointestinal (GI) tract account for approximately 4% [5]. Despite being rare, orbital metastases are the most common malignancies encountered in the orbital region. The incidence of intraocular metastasis is considerably higher than orbital metastasis itself [1, 5].

The most common symptoms of orbital metastasis include diplopia (48%), pain (19%) and gradual visual loss (16%). Clinically, they manifest as proptosis, altered eye motility, ptosis, conjunctival chemosis, and enophthalmos [1, 6]. In our patient, the presenting complaints included periorbital region pain and altered eye movement predominantly on the left side.

The pathophysiology of rectal carcinoma metastasizing to the orbit is still being studied. A possible explanation of the tumor spread is via the tumor emboli through the middle or inferior haemorrhoidal veins to the inferior vena cava, followed by pulmonary circulation and carotid arteries to finally enter the ophthalmic artery [2]. For this reason, by the time tumor emboli reaches the orbit it has already metastasized to the lungs. Our patient also had pulmonary metastases prior to orbital metastases. Another mechanism of tumor spread can be through seeding into the Batson’s venous plexus that reaches the cerebral venous sinuses and enters the ophthalmic vein [7].

Orbital metastases of rectal malignancies have a poor prognosis secondary to widespread visceral metastases by the time it reaches the orbit. The main goal of therapy in such patients is palliative care and pain management. In our case too the patient is undergoing palliative radiotherapy for symptomatic management.

## Conclusions

Orbital metastases, being extremely rare in colorectal malignancies, pose a diagnostic dilemma. Correlating between the clinical presentation and imaging findings is most helpful in its timely diagnosis. Before reaching the orbit, the primary tumor has already metastasized to multiple sites, like in our case. For this reason, orbital metastases have a very poor prognosis. Although the treatment of metastatic orbital tumors is mainly palliative, it often requires a combination of radiotherapy, chemotherapy and surgery.

Through this case presentation, we have aimed at highlighting the importance of early diagnosis of orbital metastasis secondary to colorectal malignancies. This can aid in good treatment outcomes in terms of relapse, improved quality of life and vision preservation for patients. 
